# A Numerical Study on Carbon-Fiber-Reinforced Composite Cylindrical Skirts for Solid Propeller Rockets

**DOI:** 10.3390/polym15040908

**Published:** 2023-02-11

**Authors:** Ferdinando Baldieri, Emanuele Martelli, Aniello Riccio

**Affiliations:** Department of Engineering, University of Campania “L. Vanvitelli”, Via Roma 29, 81031 Aversa, Italy

**Keywords:** solid propeller rocket, space, composite materials, carbon-fiber-reinforced polymers (CFRP), buckling, laminate strength

## Abstract

A solid rocket motor (SRM) is a rocket engine that uses a fuel/oxidizer mixture in a solid state; the most commonly employed propellants are Hydroxyl-Terminated Polybutadiene (HTPB) as the fuel and ammonium/potassium perchlorate as the oxidizer. To increase the flight range of this kind of vehicle, the weight has to be reduced as much as possible. A possible element that can be worked on is the coating of the combustion chamber: the skirt. The aim of this paper is to investigate the behavior of a cylindrical skirt subjected to internal pressure load and axial thrust and to compare the performance of a skirt made of a standard steel for aeronautics purposes with a carbon-fiber-reinforced composite skirt. The motor test case is taken from the ONERA C1xb and the flowfield is simulated with an axisymmetric *k*-*ω* turbulence model. The carbon-fiber-reinforced composite skirt is a cylindrical shell with a symmetric and balanced layup [90/0/45/−45]s. To check composite layer integrity, Hashin’s failure criteria were adopted while linearized buckling methods were used to assess the buckling behavior of the skirt. The composite layup was modeled by adopting the classical laminate theory.

## 1. Introduction

The weight reduction and greater reliability of systems in space missions is a critical issue [1]. The skirt is one of the potential elements for total weight reduction in space rocket motors, so the implementation of new configurations with advanced composite materials is desirable. Actually, the use of carbon-fiber-reinforced composite materials in aerospace industry applications, thanks to their high stiffness and strength-to-weight ratio compared to metallic alloys, is well-established [2].

Indeed, carbon-fiber-reinforced composite skirts are, potentially, easier to manufacture and assemble if compared to conventional metallic ones. Moreover, it is possible to increase the weight reduction achievable with carbon-fiber-reinforced composite materials by properly selecting the laminate stacking sequence [3]. Through the years, many studies have been performed on the manufacturing processes for skirts. In 1991, Peters et al. [4] described the filament winding process to produce composite rocket motors. According to the filament winding process, a rotating preform is adopted to lay down resin-impregnated fabric fibers. In 1985, Onoda [5], by introducing proper numerical models, searched for the best configurations for a laminated cylindrical shell under axial load, in terms of the lamination’s parameters. Other configurations with Kevlar fibers have been studied by Hoffman [6] and Niharika et al. [7]. Smerdov [8,9] described an optimization problem for cylindrical shells under axial compression and external pressure taking into account the buckling phenomenon.

After 1995, thanks to new numerical methodologies and the increasing performance of calculators, a lot of studies based on genetic algorithms and the optimization of carbon-fiber-reinforced composite material layup, finalized for weight reduction and buckling mitigation, have been presented. Actually, genetic algorithms, which are search and optimization algorithms based on the principles of natural selection [10], have been extensively adopted. Genetic algorithms are optimization algorithms generally based on the selection and manipulation of individual configurations for the production of next-generation configurations by crossover and mutation techniques [11]. This type of optimization algorithm was demonstrated to be suitable for carbon-fiber-reinforced laminates [12]. fiber-reinforced composite materials have a range of related literature about the material’s mechanical properties and applications in various scientific areas. One of those materials is glass-fiber-reinforced polymers (GFRP): they are employed as hollow composite reinforced sections (HCRSs) to contain the inner concrete wall in HCCs and as a corrosion-reduction measure [13]. They are employed in the naval field for boat-approaching slabs in addition to increasing worker efficiency during handling and installation and material durability [14]. Recent studies have proposed a new application format for these kinds of materials in the railway industry, with sleepers for mainline railway tracks reinforced with GFRP [15].

The choice of carbon fibers was made on the basis of the good compromise of their mechanical properties and ease of manufacturing and implementation. Carbon fibers with low modulus and high strength are used as filler materials, and carbon fibers with high modulus and high strength are used as reinforcement in polymeric composites. Additionally, the electrical and thermal conductivities of carbon fibers are tolerable. One of the most important advantages of using these fibers in composite materials is their enhanced fatigue resistance. Carbon fibers do not experience stress rupture, in contrast to aramid or glass fibers, although they do experience full elastic recovery after unloading [16]. Geier et al. showed that when designing laminates, it is important to monitor the effects of both the orientation of the plies and their placement inside the laminate. The common technique of fixing in advance the orientations of plies and their relative position, then optimizing only their thicknesses, is insufficient for non-symmetric laminates where membrane-bending coupling stiffnesses are involved [17]. For those reasons, the stacking sequence that was chosen for this study is a symmetric quasi-isotropic laminate [90/0/45/−45]s. The aim of this paper is to numerically test the strength and buckling characteristics of a cylindrical carbon-fiber-reinforced composite skirt, subjected to the internal pressure field and axial thrust, and to compare it with a classical metallic skirt in terms of mechanical response and weight. The motor test case taken into consideration in this paper is the scaled ONERA C1xb which is easily replicable in the laboratory. This engine was meticulously constructed (in connection with the development of the Ariane 5 P230 solid rocket motor) to examine the relationship between periodic vortex shedding and pressure oscillations, and numerous firing tests were carried out while taking into account a variety of composite propellants with variable mass percentages and particle sizes [18].

In Section 2, a description of the background mathematical models is provided. The RANS approach to fluid dynamics problems is briefly presented, and the classic lamination theory, together with the failure criteria adopted to simulate the mechanical behavior and predict the strength of composite structures, is introduced.

In Section 3, the numerical model of the ONERA C1xb motor is detailed together with the relevant boundary conditions for CFD analysis, performed within the Ansys Fluent environment, and the Finite Element Method-based structural buckling analysis, performed with the adoption of the Abaqus code. In this section, a sensitivity analysis of the mesh adopted for the cylindrical skirt is also presented.

Finally, in Section 4, results from the numerical analyses are presented and assessed.

In comparison with the studies available in the literature, this study aims to improve the methodology and approach to composite materials applications in the aerospace field. The pressure and thrust loads used as input in FEM analyses are entirely computed from CFD analyses starting from operative conditions. Those loads are used to determine the mechanical and buckling strength of a composite material skirt and the possible occurrence of damage initiation. This kind of approach can lead to a more complete and specific analysis of composite materials employed in engineering fields, with thorough numerical investigations about fluid dynamic behavior that interacts with the mechanical response. This approach is substantially different from [7,19], which do not take into account the CFD solution, and [20,21] which use a decoupled-approach CFD-FEM to optimize layer thickness.

This paper aims to verify the reliability of composite material skirts, starting from operating conditions numerically analyzed for a solid rocket motor, and shows the comparison in terms of mechanical and buckling strength with a skirt made of steel. Despite the works cited above, the composite material considered is a symmetric quasi-isotropic of carbon-fiber-reinforced plastics and the loads applied are pressure and thrust from CFD computations. Furthermore, the paper has a significant margin of improvement in mechanical strength, light weight, and thermal strength, coupling the temperature field from CFD with FEM analyses. The latter point is well-known in the literature as an improvement of mechanical finite element analyses [22].

## 2. Theoretical Background

### 2.1. Reynolds-Averaged Navier–Stokes Approach for the Flowfield

In the first instance, the internal flowfield was solved with a RANS approach in order to know the pressure load alongside the combustion chamber. This approach consists of decomposing the generic instantaneous variable in its mean value and its fluctuation with the Favre decomposition technique.

Hence, the set of equations for a compressible, unsteady, and turbulent flow to be solved can be expressed as [23,24]:

(1) $$\frac{\partial \bar{\rho }}{\partial t}+\frac{\partial }{\partial x_{j}}\;\left(\bar{\rho }\;{\tilde{u}}_{j}\right)=0$$

(2) $$\frac{\partial \left(\bar{\rho }{\tilde{u}}_{i}\right)}{\partial t}+\frac{\partial }{\partial x_{j}}\;\left({\tilde{u}}_{j}\bar{\rho }\;{\tilde{u}}_{i}\right)=-\frac{\partial p}{\partial x_{i}}+\frac{\partial \bar{\sigma_{ij}}}{\partial x_{j}}+\frac{\partial \tau_{ij}}{\partial x_{j}}$$

(3) $$\frac{\partial \left(\bar{\rho }\tilde{E}\right)}{\partial t}+\frac{\partial }{\partial x_{j}}\;\left({\tilde{u}}_{j}\bar{\rho }\;\tilde{H}\right)=\frac{\partial }{\partial x_{j}}\left(\bar{\sigma_{ij}}{\tilde{\;u}}_{i}+\bar{\sigma_{ij}u_{i}^{\prime \prime }}\right)-\frac{\partial }{\partial x_{j}}\left({\bar{q}}_{j}+c_{p}\bar{\rho u_{j}^{\prime \prime }T^{\prime \prime }}-{\tilde{u}}_{i}\tau_{ij}+\frac{1}{2}\bar{\rho u_{i}^{\prime \prime }u_{i}^{\prime \prime }u_{j}^{\prime \prime }}\right)$$

where $\bar{\rho },\;\;\bar{\rho }{\tilde{u}}_{i}$, and $\bar{\rho }\tilde{E}$ are density, momentum, and energy with Favre decomposition, respectively; $\tilde{H}=\tilde{E}+\bar{p}/\bar{\rho }$ and ${\bar{q}}_{j}\approx -\frac{c_{p}\tilde{\mu }}{Pr}\;\frac{\partial \tilde{T}}{\partial x_{j}}$. Note that $\tilde{\mu }$ is the dynamic viscosity computed using Sutherland’s Law and *Pr* is the Prandtl number [25]. The viscous stress tensor is:

(4) $${\bar{\sigma }}_{ij}\approx 2\tilde{\mu }\;\left({\tilde{S}}_{ij}-\frac{1}{3}\;\frac{\partial {\tilde{u}}_{k}}{\partial x_{k}}\;\delta_{ij}\right)$$

where ${\tilde{S}}_{ij}=\left(\partial {\tilde{u}}_{i}/\partial x_{j}+\partial {\tilde{u}}_{j}/\partial x_{i}\right)/2$. The other terms in Equation (3) need to be defined; in particular, the Reynolds stress terms can be expressed by the Boussinesq approximation:

(5) $$\tau_{ij}=2{\tilde{\mu }}_{t}\;\left({\tilde{S}}_{ij}-\frac{1}{3}\;\frac{\partial {\tilde{u}}_{k}}{\partial x_{k}}\;\delta_{ij}\right)-\frac{2}{3}\;\bar{\rho }k\;\delta_{ij}$$

The turbulent heat flux and the terms associated with molecular diffusion and turbulent transport can be represented by the following expressions:

(6) $$c_{p}\bar{\rho u_{j}^{\prime \prime }T^{\prime \prime }}\;\approx -\frac{c_{p}{\tilde{\mu }}_{t}}{Pr}\;\frac{\partial \tilde{T}}{\partial x_{j}}$$

(7) $$\bar{\sigma_{ij}u_{i}^{\prime \prime }}-\frac{1}{2}\bar{\rho u_{i}^{\prime \prime }u_{i}^{\prime \prime }u_{j}^{\prime \prime }}\;\approx \;\left(\tilde{\mu }+\frac{{\tilde{\mu }}_{t}}{\sigma_{k}}\right)\;\frac{\partial k}{\partial x_{j}}$$

Note that $Pr_{t}$ is a turbulent Prandtl number, assumed as a constant, and $\sigma_{k}$ is a coefficient. The most relevant variables are the eddy viscosity from the turbulence model $\left({\tilde{\mu }}_{t}\right)$ and the turbulent kinetic energy (k), defined as $k=\left(\bar{u_{i}^{\prime }u_{i}^{\prime }}\right)/2$.

In this work, the two-equations turbulence model *k-ω* was considered; hence, the eddy viscosity can be defined as follows [3]:

(8) $${\tilde{\mu }}_{t}=\bar{\rho }\;\frac{k}{\omega }$$

This detailed model of the gas phase allows us to correctly predict the pressure field developed by a transitional flow alongside the combustion chamber in ideal conditions without going through the chemical kinetics and complex instability phenomena of the flow. In order to catch the thermodynamic properties relevant to this study, fully turbulent conditions of the flow were imposed with Favre-averaged equations [26].

### 2.2. Lamination Theory for a Fiber-Reinforced Composite Cylindrical Skirt

During the flight, different loads act on the solid rocket motors. The most critical loading conditions are related to the axial compression *F* due to thrust and the torque *T* due to aerodynamic forces on the fins. These loads must be sustained by the cylindrical skirt. Under certain critical conditions, these loads can induce buckling and strength failure; this is the reason why the dimensioning of the skirt becomes of primary relevance for the structural integrity of the rocket. In order to study the mechanical behavior of composite skirts, the classical laminate theory for thin shells can be successfully employed.

**Figure 1:**
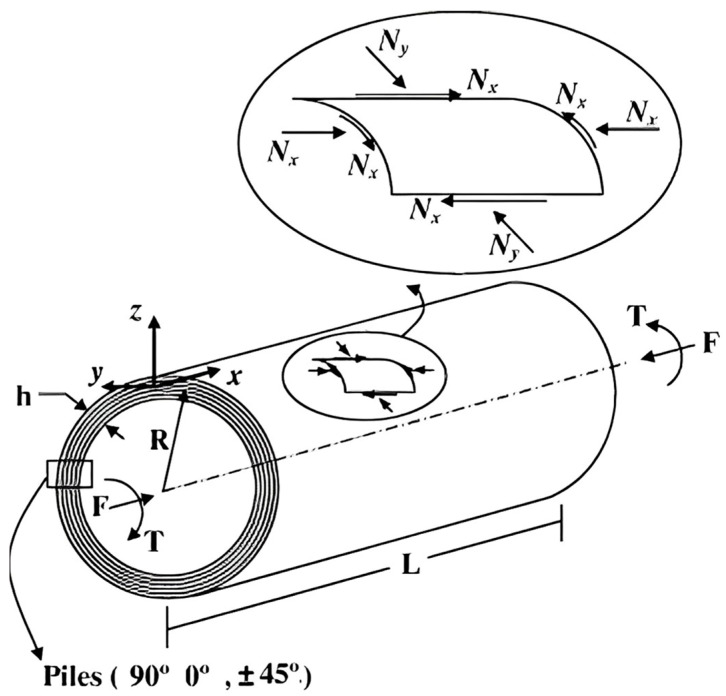
The shell elements and resultant forces of the cylindrical skirt.

For the carbon-fiber-reinforced composite cylindrical skirt of length *L*, radius *R*, and thickness *h*, shown in Figure 1, under axial compression F and aerodynamic torque *T*, the effects of aerodynamic torque can be neglected, as demonstrated by Liang and Cheng [20]. The composite laminate, adopted for the skirt, was chosen to be symmetric and balanced with respect to its mid-surface with the basic eight plies of quasi-isotropic building blocks characterized by the stacking sequence [90/0/45/−45]s (the fiber orientations of the plies being defined as the angles between the fiber direction and the load axis). The resultant force applied to the carbon-fiber-reinforced cylindrical skirt is:

(9) $$N_{x}=\frac{F}{2\pi R}$$

(10) $$N_{y}=N_{xy}=M_{x}=M_{y}=M_{xy}=0$$

(11) $$\left\{\begin{array}{c} N\\ M \end{array}\right\}=\left[\begin{array}{cc} A & B\\ B & D \end{array}\right]\left\{\begin{array}{c} \varepsilon \\ k \end{array}\right\}$$

where ε and κ are, respectively, the strain and the curvature.

Let *N* be the number of plies; the elements in the extensional stiffness matrix (*A*), the bending–extensional coupling stiffness matrix (*B*), and the bending stiffness matrix (*D*) are:

(12) $$A_{ij}=\overset{N}{\underset{k=1}{\sum }}{\bar{Q}}_{ij}^{\left(k\right)}\;\left(z_{k+1}-z_{k}\right)$$

(13) $$B_{ij}=\frac{1}{2}\overset{N}{\underset{k=1}{\sum }}{\bar{Q}}_{ij}^{\left(k\right)}\;\left(z_{k+1}^{2}-z_{k}^{2}\right)$$

(14) $$D_{ij}=\frac{1}{3}\overset{N}{\underset{k=1}{\sum }}{\bar{Q}}_{ij}^{\left(k\right)}\;\left(z_{k+1}^{3}-z_{k}^{3}\right)$$

the difference in brackets is the thickness of a single ply and ${\bar{Q}}_{ij}^{\left(k\right)}$ is the transformed reduced stiffness matrix in the global laminate coordinate system. Due to the symmetry of the laminate with respect to the mid-surface, the bending–coupling stiffness matrix [*B*] can be neglected. For these reasons, when the thrust load is applied, the constitutive equations can be written as follows:

(15) $$\left\{\begin{array}{c} N\\ M \end{array}\right\}=\left[\begin{array}{cc} A & 0\\ 0 & D \end{array}\right]\left\{\begin{array}{c} \varepsilon \\ k \end{array}\right\}$$

### 2.3. Failure Criteria for Uniform Lateral Pressure Load

As the grain burns, the pressure in the combustion chamber grows up to a constant value, $P_{c}$. The purpose of this work is to estimate the capability of steel and carbon-fiber-reinforced composite skirts to withstand failure due to overstressing.

For the skirt made of steel, the equivalent von Mises stress criterion is employed [27]; the final equation for equivalent stress, the equivalent von Mises stress criterion, can be written:

(16) $$\sigma_{EMS}=\sqrt{\frac{1}{2}\left[{\left(\sigma_{xx}-\sigma_{yy}\right)}^{2}+{\left(\sigma_{yy}-\sigma_{zz}\right)}^{2}+{\left(\sigma_{zz}-\sigma_{xx}\right)}^{2}+6\left(\tau_{xy}^{2}+\tau_{yz}^{2}+\tau_{zx}^{2}\right)\right]}$$

where $\sigma_{ii}$ and $\tau_{ij}$ are the components of the stress tensor. If the value of $\sigma_{EMS}$ is locally bigger than the maximum allowable yield stress for a given material, failure occurs.

For the carbon-fiber-reinforced composite skirt, Hashin’s failure criteria are employed. According to Hashin’s failure criteria, four different modes of failure for a composite laminate are considered: namely, fiber tension, fiber compression, matrix tension, and matrix compression [28].

Fiber tension:

(17) $${\left(\frac{\sigma_{11}}{X_{T}}\right)}^{2}+\frac{\sigma_{12}^{2}+\sigma_{13}^{2}}{S_{12}^{2}}=k$$

Fiber compression:

(18) $${\left(\frac{\sigma_{11}}{X_{C}}\right)}^{2}=k$$

Matrix tension:

(19) $$\frac{{\left(\sigma_{22}+\sigma_{33}\right)}^{2}}{Y_{T}^{2}}+\frac{\sigma_{23}^{2}-\sigma_{22}\sigma_{33}}{S_{23}^{2}}+\frac{\sigma_{12}^{2}+\sigma_{13}^{2}}{S_{12}^{2}}=k$$

Matrix compression:

(20) $$\left[{\left(\frac{Y_{C}}{2S_{23}}\right)}^{2}-1\right]\left(\frac{\sigma_{22}+\sigma_{33}}{Y_{C}}\right)+\frac{{\left(\sigma_{22}+\sigma_{33}\right)}^{2}}{4S_{23}^{2}}+\frac{\sigma_{23}^{2}-\sigma_{22}\sigma_{33}}{S_{23}^{2}}+\frac{\sigma_{12}^{2}+\sigma_{13}^{2}}{S_{12}^{2}}=k$$

where $\sigma_{ij}$ represents the stress components, $X_{T}\;$ and $Y_{T}$ are the allowable tensile strengths, $X_{C}\;$ and $Y_{C}$ are the allowable compressive strengths, and $S_{12}$, $S_{13}$, and $S_{23}$ are the allowable shear strengths. If k<1, no failure is detected, while if k ≥1, the ply or fiber and/or matrix failure occurs.

## 3. Test Case—Model Definition

**Figure 2:**
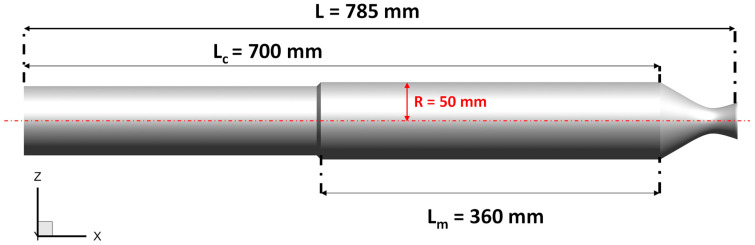
ONERA C1xb—Dimensions.

**Figure 3:**
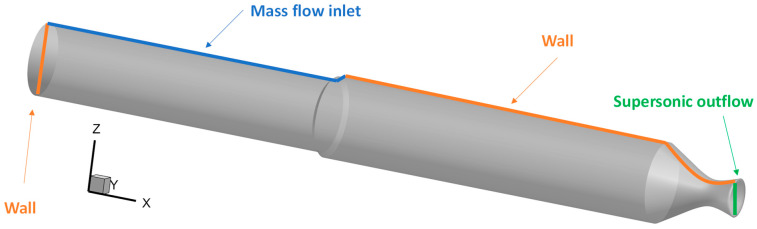
Boundary condition locations.

The selected test case is based on the laboratory-scale ONERA C1xb solid rocket motor, reproducing a firing time corresponding to a 3 mm-grain burned layer [29]. It has a length of *L* = 0.785 m and the diameter of the rocket chamber is *R* = 0.05 m, as shown in Figure 2. A transient axisymmetric simulation was performed in Ansys Fluent with the turbulence model *k-ω*, as discussed in Section 2.1, with a timestep of $2\times 10^{-6}$ s and a total timespan of T=0.116 s. This study only considers single-phase ideal gas flow computations, without the addition of aluminum particles because, at this stage, detailed chemical kinetics coupled with fluid dynamics would not considerably affect the results, besides the fact that the computational costs would be considerable. Furthermore, this level of detail is not relevant to fulfill the purpose of obtaining a suitable skirt substitution with CFRP materials. In addition, the igniter at the SRM head end is not considered [30]. The computational domain is a structured grid $N_{x}\times N_{y}\times N_{z}=140\times 20\times 100$ for a total number of 280,000 cells. A refinement near walls and grain is used to better predict the viscous effects of the mass injection of propellant. An implicit time integration to the second order of accuracy is performed, and numerical flux discretization is assessed with a second-order upwind method. The boundary conditions used for ONERA C1xb are reported in Table 1 [18], where $\rho_{p}$ is the propellant density, $V_{c}\;$ is the propellant burning rate, $\dot{m}$ is the injection mass flow rate, $T_{f}$ is the flame temperature, *c* is the speed of sound, *R* is the perfect gas constant, μ is the dynamic viscosity, γ is the ratio of specific heats, and *Pr* is the Prandtl number. As shown in Figure 3, at the injection walls, a constant mass flow rate and temperature were imposed, whereas the walls were considered adiabatic with no-slip conditions. Since the outflow is supersonic, no thermodynamic properties need to be prescribed.

The resultant pressure field alongside the combustion chamber, which is the output for the Ansys Fluent simulation, is used as an input load for the structural analysis, performed in Abaqus, to evaluate the strength and integrity of the cylindrical skirt. For this study, the nozzle part was not taken into account. The skirt was modeled as a 3D cylindrical shell body with a constant radius *R*, length $L_{c}$ (ref. Figure 2) and a thickness of 3.684 mm. The external layer of the SRM shell has a radius of 6.842 mm. S8R Abaqus elements with six degrees of freedom and eight-node quadratic doubly thick curved shell elements with a reduced integration scheme were adopted. The structural analyses, in spite of previous ones, were assessed with a three-dimensional layout in order to catch instability phenomena that could be triggered on the body, such as buckling.

The first analyzed configuration is a cylindrical skirt made of ASTM-36, standard steel for aerospace applications. ASTM-36’s mechanical properties are listed in Table 2.

The second analyzed configuration is a carbon-fiber-reinforced composite skirt made of layers of carbon/epoxy with a symmetrically balanced stacking sequence [90/0/45/−45]s following the work described in [20]; different fabrics were stacked together according to the proposed stacking sequence. In particular, each orientation was obtained by using two 0.23 mm-thick fabrics. The layer properties are listed in Table 3.

In order to assess the intra-laminar damage, the Hashin failure criteria were adopted.

The mass of the skirt is 6.36 kg and 1.29 kg, for steel ASTM-36 and the composite configuration, respectively.

**Figure 4:**
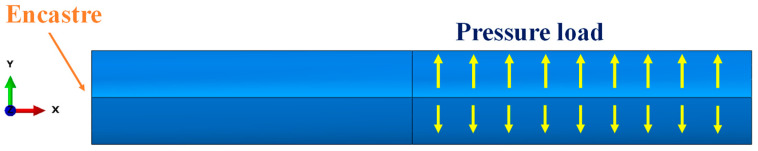
Boundary conditions on Abaqus/standard.

The solution, in terms of pressure distribution acting on the skirt’s internal surface, from Ansys Fluent code, was set as a pressure load input in Abaqus structural analysis. Since the combustion chamber is the most exposed surface to the flames and flow coming from the burning grain, the pressure load was applied only in this zone, as shown in Figure 4. The head end was considered fully constrained, to obtain the most conservative solution in terms of stress distribution.

### Mesh Sensitivity Analysis

**Figure 5:**
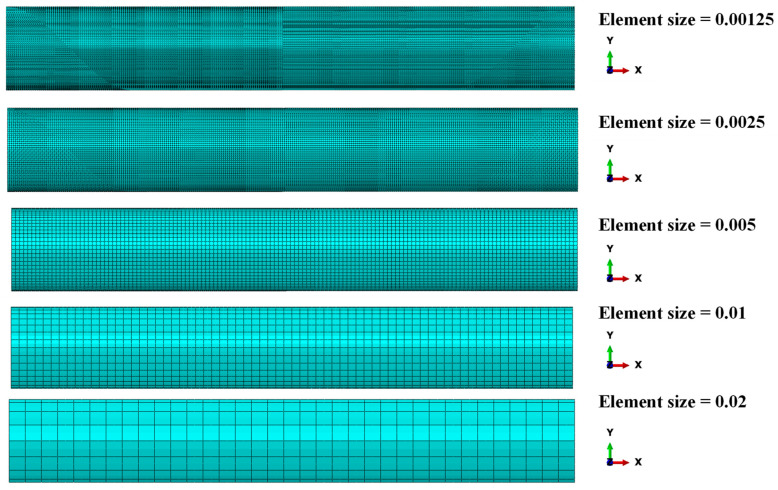
Element size S8R (mm).

**Figure 6:**
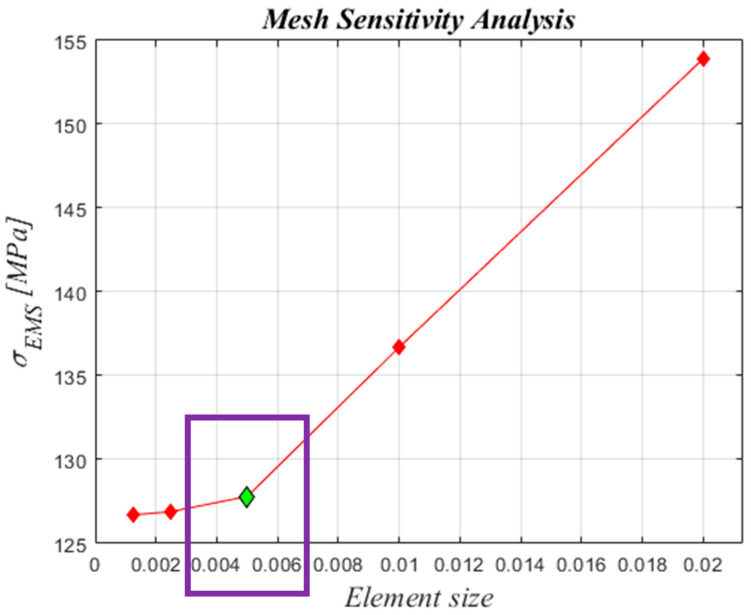
Mesh sensitivity analysis (ES expressed in (mm))—maximum equivalent von Mises stress. Chosen ES, 0.005 mm (in green).

**Figure 7:**
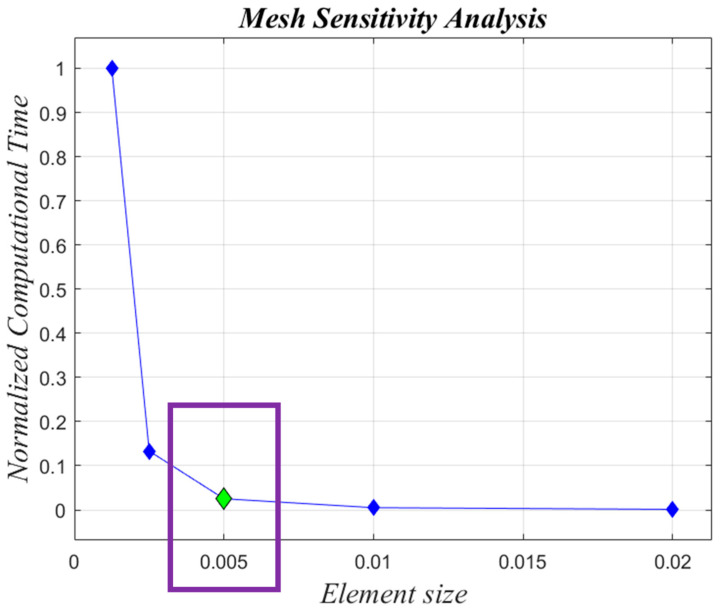
Mesh sensitivity analysis (ES expressed in (mm))—normalized computational times. Chosen ES, 0.005 mm (in green).

In order to guarantee accurate numerical results from the structural analysis, a mesh sensitivity analysis was performed on the skirt model to correctly define the size of the shell elements. The computed values of the maximum equivalent von Mises stress, for models with different shell elements sizes, were compared. The boundary conditions reported in Figure 4 were applied for the mesh sensitivity analysis. As shown in Figure 5, five different sizes of the shell elements were considered (ES = 0.00125 mm, 0.0025 mm, 0.005 mm, 0.01 mm, 0.02 mm). Figure 6 relates the maximum equivalent von Mises stress for the five different element sizes investigated. Figure 7 shows the comparison of normalized computational time for each configuration investigated. Normalization was performed with respect to the higher computational time. In Figure 6, it can be observed that simulations with ES = 0.00125 mm, 0.0025 mm, and 0.005 mm have a very similar trend, while in Figure 7, the configuration with ES = 0.005 mm shows a computational time 97% lower if compared to the ones found for the configuration with ES = 0.00125. A reasonable trade-off between computational cost and the accuracy of numerical results led us to choose ES = 0.005 mm for shell elements of both steel ASTM-36 and Carbon/Epoxy skirts.

## 4. Test Case—Numerical Results

**Figure 8:**
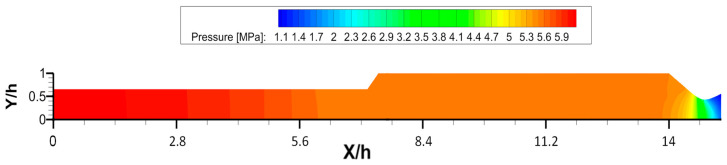
Pressure field in ONERA C1xb. In orange, the pressure contour in the combustion chamber.

From the surface of the burning grain, in the solid rocket motor ONERA C1xb, there is a mass flow injected into the chamber constantly accelerated toward the nozzle, where supersonic conditions are reached. Considering the regression rate, a firing time of T=0.166 s, which matches 3 mm of the layer of grain burned, is considered enough to guarantee a uniformly distributed pressure in the chamber. Due to the axisymmetric RANS approach, just an average mean flowfield can be displayed, which means that the inhomogeneity of the mass injection, the hydrodynamic instabilities, and the vortex shedding phenomena were not considered. Indeed, these effects were not considered of interest for the test case under consideration because they are related to non-symmetrical, non-stochastic, unpredictable, and unsteady blows in the solid rocket motor, which were not considered relevant for the period *T* under investigation. The output pressure found from the CFD analysis in the chamber is $P_{c}=5.6\;\mathrm{MPa}$, as shown in Figure 8. The simulations to assess the mechanical behaviors of the skirts were carried out in Abaqus by applying the pressure load all over the part of the skirt not coated with grain. Subsequently, a comparison between the steel ASTM-36 and carbon-fiber-reinforced skirt was carried out.

**Figure 9:**
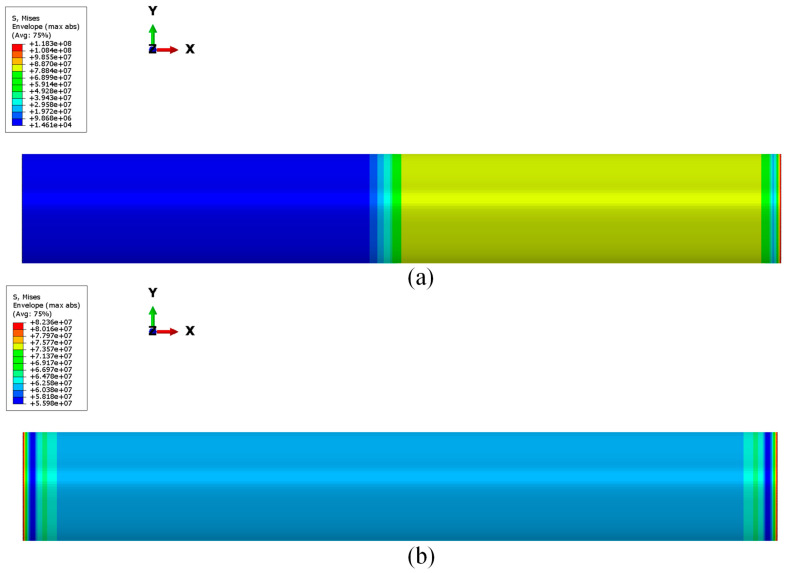
Von Mises stress (Pa) on steel skirt under pressure load (**a**) and thrust load (**b**).

In Figure 9, the contour plots of the von Mises stress distribution all over the steel skirt are shown both for pressure load and thrust. The maximum value of the von Mises stress is located on the free edge and it is lower than the yield stress. This means that there is no plastic deformation and failure does not occur under the analyzed pressure conditions.

**Figure 10:**
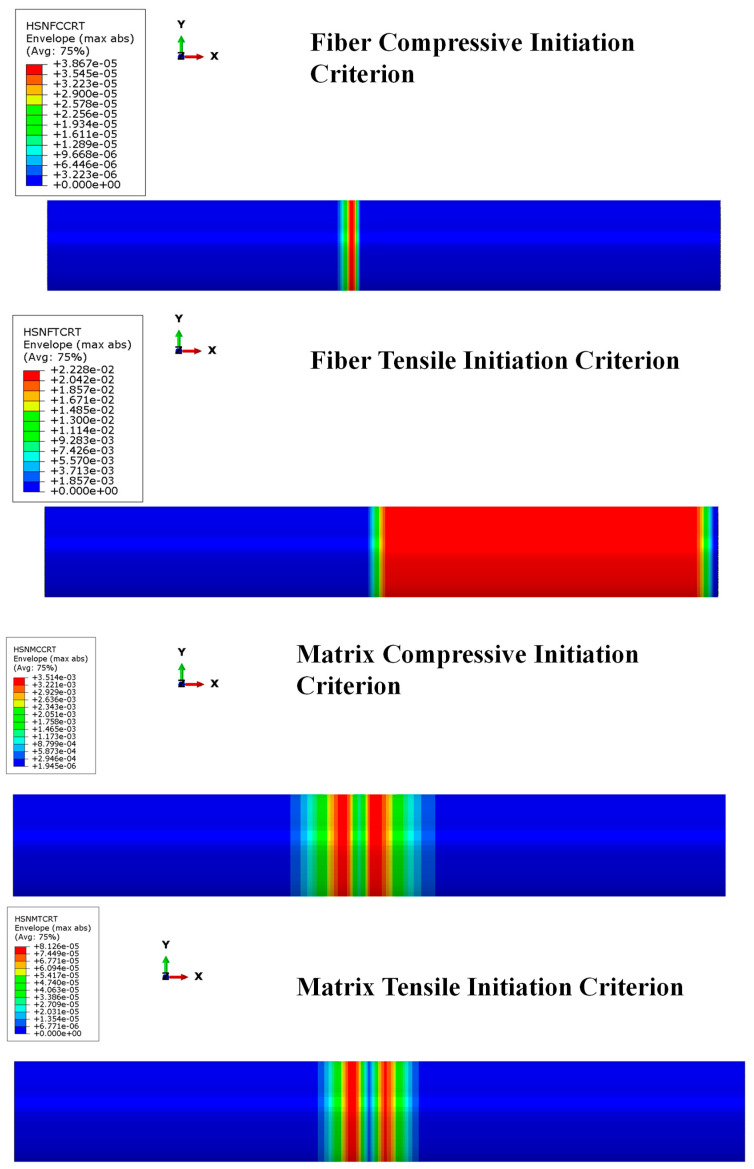
Hashin criteria: fiber and matrix compression/tensile behavior under pressure load.

**Figure 11:**
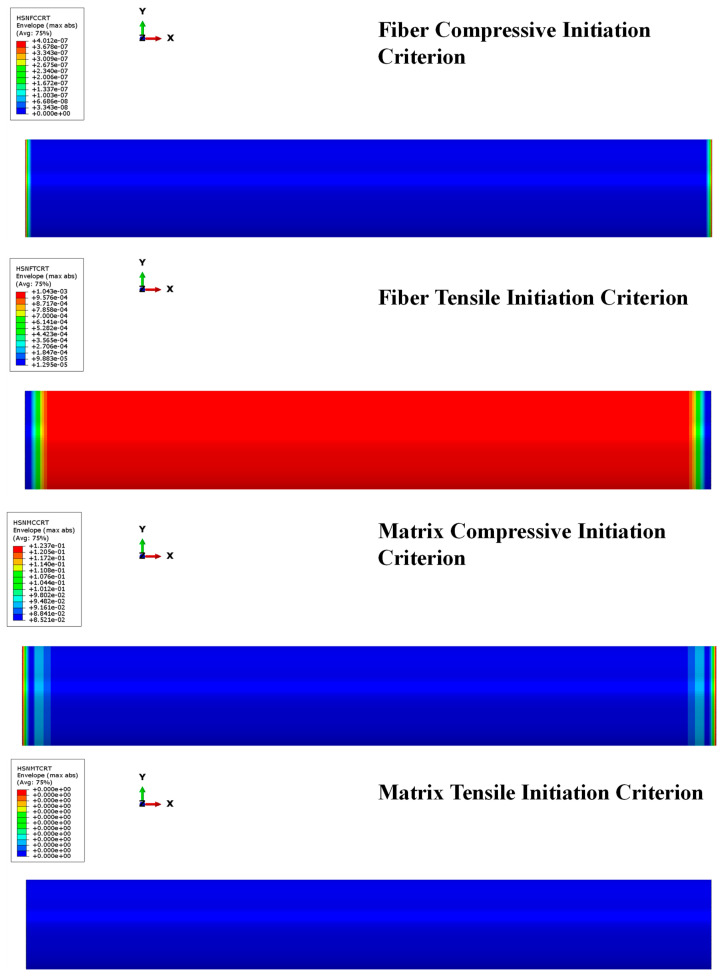
Hashin criteria: fiber and matrix compression/tensile behavior under thrust load.

Additionally, Figure 10 and Figure 11 show that the Hashin criteria are not matched for both fiber and matrix in any ply of the composite skirt by pressure and thrust loads, confirming the reliability of the composite skirt solution. The results show that the most stressed zone is the combustion chamber, as well. In that zone, the failure criteria are not matched within a wide safety margin; in fact, Hashin criteria are matched when the local values are greater than one. The matrix shows good strength both for compressive and tensile behavior as well as fibers. Since the global values returned by Hashin criteria are several orders of magnitude below unity, there are, without doubt, margins of improvement in terms of composite material skirt thickness.

**Figure 12:**
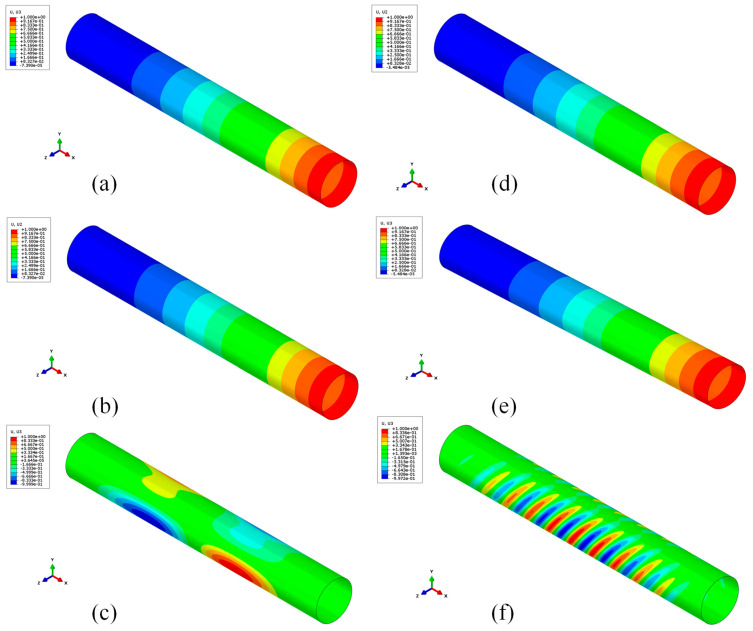
Steel configuration: Mode 1 (**a**), Mode 2 (**b**), Mode 3 (**c**). CFRP configuration: Mode 1 (**d**), Mode 2 (**e**), Mode 3 (**f**).

As already mentioned, the effect of the thrust load on the elastic instability was also assessed. The compressive load in the axial direction due to thrust generation is a critical issue for space rocket motor coatings, hence a linear buckling analysis was performed to evaluate the mechanical behavior of the investigated configurations. The buckling load applied is defined as discussed in Section 2.2, taking into account a thrust force of 70 kN. Figure 12 shows the first, second, and third buckling modes of the steel and composite skirts. Both for the steel and CFRP skirts, the first and second eigenvalues associated with Mode 1 and Mode 2 are basically the same. Eigenvalues show that for a steel skirt, the buckling instability threshold occurs for an applied load that is 27 times the current load, whereas for a fiber-reinforced composite skirt, the buckling instability threshold occurs for an applied load that is five times the current load (as reported in Table 4). For both configurations, buckling instability does not occur; however, the composite skirt configuration is able to guarantee a weight reduction from 6.36 kg to 1.29 kg. Moreover, those results show that both structures are, under pressure and thrust loads, oversized in thickness and there is a wide range of improvements needed in order to make those structures as light and durable as possible. Furthermore, a comparison between the steel and CFRP configurations was assessed, reducing the steel skirt’s thickness, in order to obtain the same buckling load. The new value of thickness was defined as 0.92 mm. A brief overview of the results is shown in Table 5. The results show that, with a similar buckling load, the steel skirt has a weight greater than the CFRP skirt. This leads us to conclude that a composite material replacement of the skirt in solid rocket motors can be affordable. Moreover, with a successive stacking sequence, and therefore thickness optimization, a lightweight configuration can be achieved, with very good properties in terms of buckling load and mechanical strength.

## 5. Conclusions

Minimizing the weight of a solid rocket motor is a critical issue for space missions. A key role is played by the skirts that cover the internal systems. The test case used for investigations was the ONERA C1xb laboratory motor. A CFD simulation was carried out with a k-ω turbulence model for a firing time corresponding to 3 mm of burned layers of grain in order to evaluate the value of the pressure field in the chamber, which was found to be equal to 5.6 MPa. This value was used as input for Finite Element structural analyses to investigate the strength and the buckling of steel and carbon-fiber-reinforced composite skirts. The temperature influence and the heating of the SRM were not taken into account on the shells’ stress–strain states for both steel and CFRP materials because of the adiabatic condition imposed on the wall; that was the only available option to obtain the most conservative solution since there are ablation phenomena of coating materials not taken into account for this study. These could be features to consider for possible future developments. Actually, a comparison was made between a common steel skirt configuration used for aerospace applications and a carbon-fiber-reinforced composite skirt with a balanced and symmetrical stacking sequence [90/0/45/−45]s.

The results demonstrated that the carbon-fiber-reinforced skirt has good strength and buckling characteristics. The carbon-reinforced composite skirt is able to guarantee a reduction of about 80% of the weight if compared with the steel configuration.

Since the Hashin criteria both for matrix and fibers showed that no failure occurs, as there are values with several orders of magnitude below unity, it is actually possible to complete composite material skirt manufacturing with a gain in terms of weight reduction. These results show that a further reduction in skirt thickness is possible and optimizable by means of, for example, multi-objective optimization.

A possible future development could be the optimization of the stacking sequence to find the best solution to further improve the strength and buckling behavior.

In light of a possible further thickness reduction (and, consequently, a weight minimization), future developments reside in the parametrization of fiber orientation in order to obtain an overall composite structure with good strength performance at as light a weight as possible. Carbon-fiber-reinforced carbon (CFRC) materials could also be employed to test structural strength and thermal resistance simultaneously. In order to validate the numerical results, prototypes modeled with additive manufacturing techniques can also be produced.

## Figures and Tables

**Table 1:** Boundary conditions for ONERA C1xb test case [18].

Property	Value
$\rho_{p}\;\left[\frac{\mathrm{kg}}{m^{3}}\right]$	1586
$V_{c}\;\left[m/s\right]$	4.18$\times 10^{-3}$
$\dot{m}\;\left(\mathrm{kg}/s/m^{2}\right)\;$	6.62948
$T_{f}\;\left(K\right)$	2224
*c* (m/s)	1031
*R* (J/kg/K)	384.513
μ (kg/m/s)	$7\times 10^{-5}$
γ	1.243
*Pr*	0.495

**Table 2:** Elastic and plastic properties of steel ASTM-36 [31].

Property	Value
Density (kg/m^3^)	7850
Young’s modulus (GPa)	210
Poisson’s ratio	0.33
Yield stress (MPa)	550
Plastic strain	0

**Table 3:** Mechanical properties of carbon/epoxy [32].

Property	Value
Density (kg/m^3^)	1594.61
E_1_ (GPa)	137
E_2_ = E_3_ (GPa)	8.17
G_12_ = G_13_ (GPa)	4.75
G_23_ (GPa)	4.0
ν_12_ = ν_13_	0.3
ν_23_	0.316
Longitudinal Tensile Strength (GPa)	1.47
Longitudinal Compressive Strength (GPa)	0.98
Transverse Tensile Strength (GPa)	0.0392
Transverse Compressive Strength (GPa)	0.0784
Longitudinal Shear Strength (GPa)	0.0784
Transverse Shear Strength (GPa)	0.0784
Longitudinal Tensile Fracture Energy (kJ/m^2^)	15,773
Longitudinal Compressive Fracture Energy (kJ/m^2^)	7010.218
Transverse Tensile Fracture Energy (kJ/m^2^)	188.083
Transverse Compressive Fracture Energy (kJ/m^2^)	752.332

**Table 4:** Comparisons between steel and CFRP skirts.

	Steel	CFRP
Eigenvalue (Mode 1)	21.174	5.26
Eigenvalue (Mode 2)	21.174	5.26
Eigenvalue (Mode 3)	109.78	27.126
Weight (kg)	6.36	1.29

**Table 5:** Comparisons between steel and CFRP skirts with the same buckling load.

	CFRP	Steel (Old)	Steel (New)
Eigenvalue (Mode 1)	5.26	21.174	5.26
Weight (kg)	1.29	6.36	1.7

## Data Availability

The data that support the findings of this study are available on request from the corresponding author.
